# Potential Usefulness of Blood Urea Nitrogen to Creatinine Ratio in the Prediction and Early Detection of Delirium Motor Subtype in the Intensive Care Unit

**DOI:** 10.3390/jcm11175073

**Published:** 2022-08-29

**Authors:** Woo Rhim Park, Hye Rim Kim, Jin Young Park, Hesun Erin Kim, Jaehwa Cho, Jooyoung Oh

**Affiliations:** 1 Department of Psychiatry, Gangnam Severance Hospital, Yonsei University College of Medicine, Seoul 06273, Korea; 2 Institute of Behavioral Sciences in Medicine, Yonsei University College of Medicine, Seoul 03722, Korea; 3 Department of Biomedical Systems Informatics, Yonsei University College of Medicine, Seoul 06273, Korea; 4 Department of Psychiatry, Yongin Severance Hospital, Yonsei University College of Medicine, Yongin 16995, Korea; 5 Department of Pulmonary and Critical Care Medicine, Gangnam Severance Hospital, Yonsei University College of Medicine, Seoul 06273, Korea

**Keywords:** delirium, blood urea nitrogen to creatinine ratio, critical illness, delirium motor subtype, biomarker

## Abstract

Prediction and early detection of delirium can improve patient outcomes. A high blood urea nitrogen to creatinine ratio (BCR), which reflects dehydration, has been reported as a risk factor for delirium. Additionally, BCR represents skeletal muscle loss in intensive care unit (ICU) patients, which can have critical implications for clinical outcomes. We investigated whether BCR could be used to predict the occurrence and motor subtype of delirium in ICU patients through a retrospective cohort study that included 7167 patients (50 years or older) admitted to the ICU. Patients were assessed daily using the Richmond Agitation-Sedation Scale and the Confusion Assessment Method for ICU and categorized according to the delirium subtype. Participants were split into 10 groups according to BCR at ICU admission and the prevalence of each delirium subtype was compared. Multivariable logistic regression was then used for analysis. A higher BCR at ICU admission was associated with the development of hypoactive delirium. Moreover, BCR > 24.9 was associated with higher rates of hypoactive delirium. Our findings showed that a high BCR at ICU admission was associated with the development of hypoactive delirium, which suggested that BCR could be a potential biomarker for hypoactive delirium in ICU patients.

## 1. Introduction

Delirium commonly occurs among critically ill patients, especially older adults. It is associated with poor outcomes such as prolonged intensive care unit (ICU) stays, readmissions, and higher morbidity and mortality rates [1,2,3]. Despite the high prevalence and clinical importance of delirium in ICU patients, it is often difficult to detect and easily neglected [4,5].

Furthermore, variations in the motor phenotypes of delirium make it more difficult to detect. Hypoactive delirium, the most prevalent motor subtype of delirium, constitutes 50% of delirium, followed by mixed delirium and hyperactive delirium [6,7]. The clinical presentation of hypoactive delirium exacerbates this problem. Symptoms of hypoactive delirium include reduced activity, apathy, decreased amount or speed of speech, decreased alertness, reduced awareness, and hypersomnolence [8]. Moreover, the hypoactive motor subtype is associated with a poorer prognosis and higher mortality rates compared to the hyperactive motor subtype [6,9,10].

Since it is important to predict and quickly detect patients with delirium and rapidly correct modifiable factors, several studies have been conducted to identify risk factors for delirium. To date, a number of theories have been proposed to elucidate the development of delirium [11]. A variety of conditions have been demonstrated to be associated with delirium, including hypoxia or hyperoxia-related brain injury, neuroinflammation, tryptophan pathway dysregulation, and gut microbiota dysregulation [12]. However, its underlying pathophysiology remains unclear. Moreover, delirium development is difficult to predict due to the cumulative effect that multiple risk factors have on the condition [13,14,15]. Furthermore, the reporting of several risk factors has been controversial due to differences in ICU environments and heterogeneity in delirium phenotypes.

In the early days of delirium research, patients with clinically dehydrating symptoms were found to have high incidences of delirium. As a high blood urea nitrogen (BUN)-to-creatinine (Cr) ratio (BCR) was used as a marker to reflect this dehydrated condition, a high BCR was identified as a major predisposing factor of delirium. Specifically, BCR > 18 was suggested as an independent risk factor for delirium [16,17]. A subsequent study revealed that dehydration could contribute to delirium by causing cerebral hypoperfusion and alterations in brain neurotransmitter levels [18]. Several proposed mechanisms of delirium, including imbalance of dopamine and acetylcholine, dysregulation of cytokine and chemokine permeability, and alteration of neurotransmitter synthesis [11], are possibly related to dehydration. However, further research has not been conducted due to the difficulty in quantitatively measuring dehydration status. A previous study that identified BCR > 18 as a risk factor for delirium could not be replicated [19]. Thus, the extent of the relationship between the BCR and delirium, as well as the optimal BCR cutoff value for predicting delirium, remain unclear.

Recently, the BCR has been investigated as a measure that can reflect reduced muscle mass in patients with chronic critical illness [20,21]. It has been shown that the BCR effectively reflects decreases in lumbar muscle mass in ICU patients [21]. Skeletal muscle loss is associated with a poor prognosis, mortality, and longer hospital stays [22]. Moreover, recent studies have shown that patients with a low skeletal muscle mass have higher delirium occurrence rates; specifically, skeletal muscle loss has been linked to the hypoactive subtype of delirium [23,24]. This evidence further emphasizes the need to examine the association between each motor subtype of delirium and the BCR.

In this observational study, we aimed to evaluate the usefulness of the BCR at ICU admission in the prediction and early detection of ICU delirium using ICU big data. In addition, to examine the specific relationship between the BCR and each delirium motor subtype, we analyzed the predictive power of the BCR for each motor subtype. We hypothesized that the BCR was associated with the hypoactive subtype of delirium but not with the hyperactive or mixed subtypes.

## 2. Materials and Methods

### 2.1. Study Participants

This longitudinal observational study was conducted on critically ill patients admitted to the ICU at Gangnam Severance Hospital, a tertiary referral hospital in Seoul, Korea, from 1 October 2014 to 31 December 2020. A total of 10,913 patients who had been admitted to the ICU were enrolled in this study. Subsequently, 1380 patients with delirium and 5787 patients without delirium were selected for the study. The exclusion criteria were: (1) no recorded evaluation data or absent data at the scheduled time of evaluation; (2) age under 50; and (3) any missing data including BUN, Cr, or Acute Physiology and Chronic Health Evaluation (APACHE) II score at ICU admission date.

All patient data, including sex, age, delirium subtypes, length of ICU stays, APACHE II scores, sedative use, and medical history such as history of major surgery, kidney disease, diabetes mellitus, heart disease, stroke, and septic condition were extracted from the electronic medical records (EMRs). We only included a patient’s medical history that was present before the onset of delirium based on the International Classification of Diseases, 10th Revision (ICD-10) diagnostic codes.

**Figure 1:**
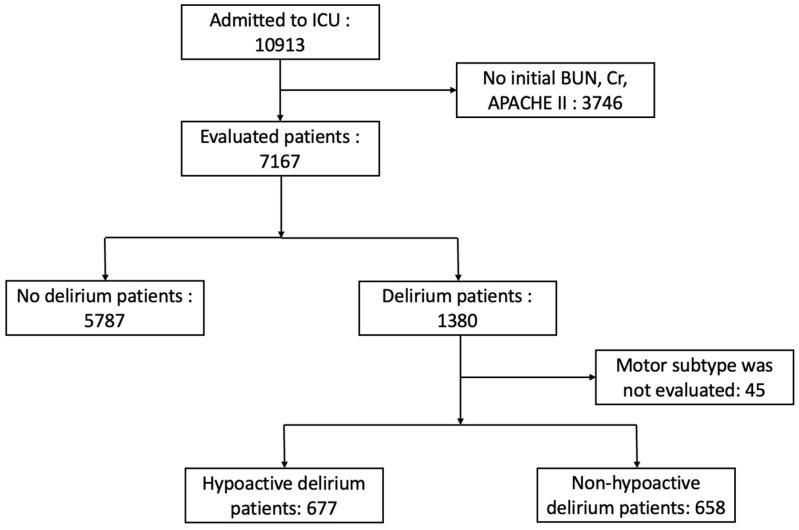
Flowchart depicting the inclusion criteria.

Patients were then divided into “Delirium” and “No Delirium” groups; if the patient had delirium symptoms at least once in ICU, the patient was included in the “Delirium” group. The Delirium group was divided into two groups based on motor subtypes; when the same patient experienced different delirium subtypes, the delirium subtype that first emerged was chosen. If the patient had the hypoactive motor subtype of delirium, the patient was classified into the “Hypoactive delirium” group; if the patient had hyperactive or mixed motor subtype of delirium, the patient was classified into the “Non-hypoactive delirium” group (Figure 1).

### 2.2. Delirium Evaluation (IDDM Protocols)

The current study was a part of the ongoing ICU Distress and Delirium Management (IDDM) project. This project has been carried out in the intensive care unit of Gangnam Severance Hospital since 2012 to closely monitor and manage the distress and delirium of ICU patients [25]. Following the protocol, nurses at the intensive care unit made rounds every day to identify and assess delirious patients. They evaluated each patients using the Richmond Agitation–Sedation Scale (RASS) and the Confusion Assessment Method for ICU (CAM-ICU). Patients who were deeply sedated (RASS score = −4 or −5) were excluded from such evaluation.

Every day at around 10 AM, a trained psychiatrist evaluated each patient, reviewed their medical chart, and determined whether the patient was delirious based on the CAM-ICU results and the Diagnostic and Statistical Manual of Mental Disorders, Fifth Edition (DSM-5). All patients over the age of 50 years were evaluated daily from the time of admission to the time of discharge. The evaluation results were recorded in the EMR.

When the mental state was found to be delirious (CAM-ICU: positive), the delirium severity and subtypes were assessed using the Korean version of the Delirium Rating Scale [26,27] and the Delirium Motor Subtype Scale (DMSS) [28,29]. While DMSS scoring requires at least two symptoms from the hyperactive or hypoactive list to meet the subtype criteria, individuals meeting the criteria for both subtypes were deemed to have the mixed subtype [27]. Ethical approval for this study was obtained from the institutional review board of Gangnam Severance Hospital, Yonsei University. (No. 3-2014-0041; date of approval: 14 April 2017).

### 2.3. Statistics

All data are presented as the mean (standard deviation (SD)) or percentages with differences between groups. Continuous variables were compared using an analysis of variance (ANOVA), whereas categorical variables were compared using a chi-squared test. The Bonferroni procedure was used during the post hoc analysis for the ANOVA.

We divided the distribution of the BCR by decile (10 equal groups) and compared the delirium prevalence in all groups. We compared the prevalence of overall delirium and each delirium motor subtype among the decile groups. The optimal cutoff value was calculated using the Youden index to discriminate the development of overall delirium and each delirium subtype.

Univariate logistic regression was performed for each variable with each delirium motor subtype. Subsequently, to determine whether the BCR acted as an independent risk factor for delirium depending on the subtype of delirium, all statistically significant and clinically valid variables were used in multiple logistic regression models for each delirium subtype. The BCR was used both as a continuous and binary variable based on its cutoff value; *p* < 0.05 was considered to be significant in all analyses. All analyses were performed using SAS (version 9.4, SAS Inc., Cary, NC, USA).

## 3. Results

### 3.1. Demographic and Clinical Characteristics

The demographic and clinical characteristics of each patient included sex, age, delirium subtype, length of ICU stay, APACHE II score, surgical status, underlying medical condition (including DM, heart disease, past stroke history, kidney disease, and sepsis), and sedative use (Table 1). The mean age of the patients was 63.6 ± 17.4 years. There were 2825 female patients (39.4%). The mean length of stay in the ICU was 7.0 ± 13.2 days. The mean APACHE II score was 17.8 ± 8.3. Prior to ICU admission, 4867 patients (68.3%) underwent surgery. During ICU stay, 5762 patients (80.9%) used sedatives.

The Delirium group patients were found to be older, stayed in the ICU for a longer period, and had significantly higher APACHE II scores than patients in the No Delirium group. Patients in the Delirium group also had significantly higher rates of underlying medical conditions such as kidney disease, diabetes, heart disease, stroke, and sepsis. The rate of sedative use was also higher in the patients in the Delirium group. BCR was found to be significantly higher in the Hypoactive delirium group compared to the No Delirium group, while there was no significant difference in BCR between the Non-hypoactive delirium group and the No Delirium group.

A total of 1380 patients (19.3%) experienced delirium during the ICU stay. A total of 677 patients (49.1%) had the hypoactive motor subtype of delirium, while 399 (28.9%) and 259 (18.8%) patients had the hyperactive and mixed motor subtypes, respectively. The average delirium onset date after ICU admission was 5.8 ± 8.5 days. The majority of patients (68.3%) were admitted to the ICU after surgery. Additional patient characteristics are presented in Table 1.

**Figure 2:**
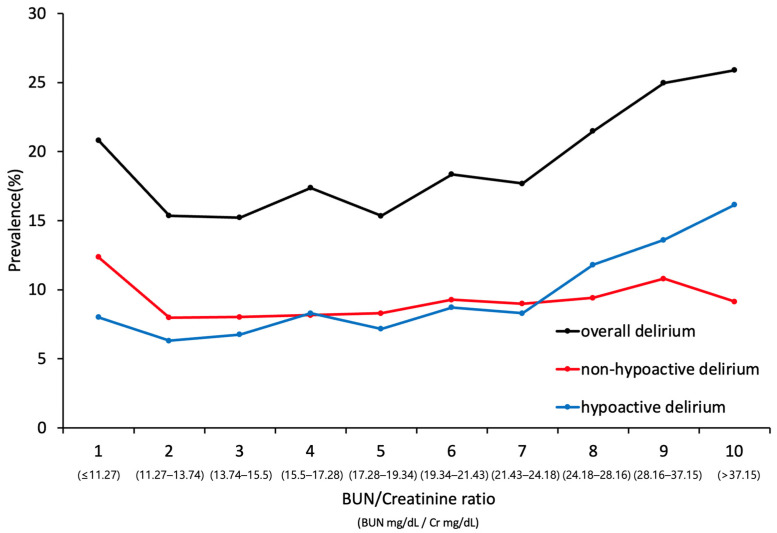
Association between prevalence of delirium subtypes and BUN/Cr ratio.

We analyzed the occurrence of delirium in each decile group as defined by the BCR in the whole set of patients. The relationship between different categories of the baseline BCR and overall delirium occurrence showed a J-shaped curve, with the lowest delirium occurrence in Decile 3 (BCR 13.7–15.4). Subsequently, we compared the incidence rate by dividing the Delirium patients into two groups according to subtype. In the Hypoactive delirium motor subtype group, the BCR was positively correlated with delirium prevalence. In contrast, in the Non-hypoactive delirium subtype group, the delirium prevalence was similar regardless of the BCR (Figure 2).

### 3.2. Multiple Logistic Regression Model

In the univariate analysis, non-hypoactive delirium was significantly associated with age (*p* < 0.0001), APACHE II score (*p* < 0.0001), kidney disease (*p* < 0.0001), diabetes mellitus (*p* = 0.0015), heart disease (*p* = 0.0223), stroke (*p* = 0.0019), sepsis (*p* < 0.0001), sedative use (*p* = 0.013), and patient type (medical) (*p* = 0.0008). Hypoactive delirium had nearly similar associations with these variables except for BCR, which showed a significant association only in hypoactive delirium (*p* < 0.0001) (Table 2).

In the multivariate logistic regression model including the above factors, age (*p* < 0.0001), APACHE II score (*p* < 0.0001), sepsis (*p* = 0.002), and sedative use (*p* = 0.0003) were identified as independent risk factors for non-hypoactive delirium. Age (*p* < 0.0001), APACHE II score (*p* < 0.0001), BCR (*p* < 0.0001), stroke (*p* < 0.0001), sepsis (*p* < 0.0001), and sedative use (*p* = 0.0023) were identified as risk factors for hypoactive delirium.

The BCR at ICU admission was associated only with hypoactive delirium development (OR 1.012; 95% CI, 1.008–1.016; *p* < 0.0001) in the multiple logistic regression model. In contrast, the BCR at ICU admission was not associated with non-hypoactive delirium development (OR 0.996; 95% CI, 0.99–1.002; *p* = 0.2167) (Table 2). The performance of each multiple logistic regression model was evaluated using the area under the receiver operating characteristic curve (AUC), which was 0.694 for non-hypoactive delirium and 0.739 for hypoactive delirium.

The optimal BCR cutoff value for hypoactive delirium, which was calculated using the Youden index, was 24.9. The odds ratio for the incidence of hypoactive delirium in patients with BCR > 24.9 was 1.677 (95% CI, 1.405-2.002; *p* < 0.0001) relative to patients with BCR < 24.9.

## 4. Discussion

In this retrospective study of 10,913 critically ill patients admitted to an intensive care unit in a tertiary care hospital, we demonstrated a significant independent correlation between a high BCR and hypoactive delirium occurrence. This association remained statistically significant even after adjustment for relevant confounders such as age, sex, APACHE II score, underlying medical conditions (kidney disease, diabetes mellitus, heart disease, stroke, and sepsis), and sedative use. Conversely, there was no association between the baseline BCR and the development of non-hypoactive delirium.

The BCR has been reported as a marker for dehydration and is thought to be associated with delirium [16]. Chronic skeletal muscle catabolism in critically ill patients has recently received considerable attention due to its association with worse outcomes, including mortality, longer hospital stays, and long-term cognitive impairment [30,31]. Particularly, the BCR was reported as a promising biomarker for chronic muscle catabolism [20,21]. Moreover, in the general population, sarcopenia was elucidated as an independent risk factor for cognitive impairment [32,33]. The ‘brain-muscle loop’ is a possible suggested mechanism, which includes cortical volume loss and white matter changes in sarcopenic patients [34]. Therefore, it is worth examining the potential significance of the BCR in the prediction and early detection of delirium and other cognitive problems.

Indeed, a low skeletal muscle mass was associated with delirium occurrence in admitted patients in previous studies [24,35]. Makiguchi et al. investigated the relationship between low skeletal muscle mass and the occurrence of hypoactive delirium in postoperative oral cancer patients [23]. Patients in their hypoactive subtype group had a significantly low muscle mass compared to patients in their no delirium group (36.9 ± 6.4 vs. 39.6 ± 8.5, *p* = 0.019). The odds ratio for skeletal muscle index (cm^2^/m^2^) in hypoactive delirium was 2.52 [23]. The researchers speculated that physically frail patients tended to have hypoactive-type delirium. We hypothesized that skeletal muscle loss in ICU patients could be reflected by the BCR and may be associated with the occurrence of hypoactive delirium. Our results supported this hypothesis.

The notable finding of this study was that the relationship between the BCR and delirium occurrence was only exhibited in patients with the hypoactive subtype. Pathophysiology of delirium, regardless of its subtype, includes neuroinflammation, oxidative stress, circadian rhythm dysregulation, tryptophan metabolism dysregulation, and gut microbiota dysregulation [12,36,37]. However, despite the different clinical presentations and outcomes, little is known about the distinct pathophysiology and related risk factors for each delirium subtype. It should be noted that the underlying mechanisms of how sarcopenia and a low BCR can contribute to hypoactive delirium were beyond the scope of this study. This study showed the association between a higher baseline BCR at ICU admission and hypoactive delirium development in an ICU patient group, which included a large spectrum of demographics and medical conditions. We assumed that the BCR had a higher chance of being linked with the core pathophysiological mechanism of hypoactive delirium.

In real-world clinical settings, despite its high prevalence in ICU patients, hypoactive delirium can be easily missed by clinicians. Moreover, hypoactive delirium is associated with worse clinical outcomes, including higher mortality and long-term cognitive sequelae [38]. Our results supported the usefulness of the BCR at ICU admission in the prediction and early detection of hypoactive delirium in ICU patients. For instance, regular assessment for hypoactive delirium in patients with a high BCR on the day of ICU admission could be emphasized.

Our study had several limitations. First, as our results were based on an observational study, the causal relationship between the BCR and delirium occurrence remains unclear. Further well-controlled studies are needed to replicate our results and confirm the causality of this relationship. Second, we did not fully consider the clinical factors that can affect delirium occurrence such as diagnosis, ventilator use, medication usage, and pre-existing dementia. However, despite not including the above delirium risk factors, our model’s predictive performance for delirium (AUC = 0.694, 0.739) was not inferior compared to the pre-existing delirium predictive model (AUC = 0.52~0.94) [39]. Lastly, this was a single-center study with limited generalizability; however, it was conducted at a university hospital that is a tertiary hospital that manages several types of ICU patients and shares characteristics with similar centers in resource-rich countries.

## 5. Conclusions

Our results indicated that a high BCR at ICU admission was associated with the development of hypoactive delirium. Our study suggested that the BCR could be a potential biomarker for the prediction and early detection of hypoactive delirium in ICU patients. As the clinical implications of the distinction of delirium subtype increases, further research will be essential for the evaluation of the distinct pathophysiology and risk factors associated with different motor subtypes of delirium.

## Figures and Tables

**Table 1:** Baseline characteristics of patients.

Variable	Class	Total (*n* = 7122)	No Delirium (*n* = 5787)	Non-Hypo (*n* = 658)	Hypo (*n* = 677)	*p*-Value	Post Hoc		
							1 vs. 2	1 vs. 3	2 vs. 3
Sex	Female	2825 (39.7%)	2277 (39.4%)	259 (40.0%)	289 (42.7%)	0.2398	>0.9999	0.2781	0.6501
Age (years)	-	63.6 (±17.4)	62.2 (±17.7)	69.6 (±14.6)	70 (±15.2)	<0.001	<0.001	<0.001	<0.001
ICU days	-	7.0 (±13.2)	4.7 (±9.9)	14.6 (±17.7)	18.3 (±21.6)	<0.001	<0.001	<0.001	<0.001
APACHE II	-	17.8 (±8.3)	16.9 (±8.1)	21.1 (±8.4)	22.1 (±8.1)	<0.001	<0.001	<0.001	0.0441
BUN (mg/dL)	-	22.5 (±19.9)	21 (±18.3)	27.9 (±22.2)	30.2 (±26.8)	<0.001	<0.001	<0.001	0.1013
Creatinine (mg/dL)	-	1.3 (±1.7)	1.2 ± 1.6	1.6 ± 1.8	1.5 ± 2	<0.001	<0.001	<0.001	0.791
BCR	-	22.9 ± 16.4	22.6 ± 16.3	22.1 ± 12.7	27 ± 20	<0.001	>0.9999	<0.001	<0.001
Kidney disease	Yes	522 (7.3%)	371 (6.4%)	70 (10.6%)	81 (12.0%)	<0.001	<0.001	<0.001	>0.9999
Diabetes mellitus	Yes	615 (8.6%)	459 (7.9%)	76 (11.6%)	80 (11.8%)	<0.001	0.0042	0.0015	>0.9999
Heart disease	Yes	528 (7.4%)	404 (7%)	62 (9.4%)	62 (9.2%)	0.0035	0.0657	0.1149	>0.9999
Stroke	Yes	216 (3%)	130 (2.2%)	28 (4.3%)	58 (8.6%)	<0.001	0.0048	<0.001	0.0039
Sepsis	Yes	363 (5.1%)	229 (4%)	56 (8.5%)	78 (11.5%)	<0.001	<0.001	<0.001	0.2016
Sedative use	Yes	5762 (80.9%)	4647 (80.3%)	555 (84.3%)	560 (82.7%)	0.0047	0.0381	0.3981	>0.9999
Patient type	Medical	2255 (31.7%)	1742 (30.1%)	240 (36.5%)	273 (40.3%)	<0.001	0.0024	<0.001	0.4443
	Surgical	4867 (68.3%)	4045 (70.0%)	418 (63.5%)	404 (59.7%)				
Delirium onset (days)		5.8 (±8.5)	-	5.2 (±7.4)	6.5 (±9.4)				
DMSS	Missing	45 (3.3%)	-	45 (3.3%)	-				
	Hyperactive	399 (28.9%)	-	399 (60.6%)	-				
	Hypoactive	677 (49.1%)	-	-	677 (100.0%)				
	Mixed	259 (18.8%)	-	259 (39.4%)	-				

Values are number (%) or mean (±standard deviation); post hoc analysis group (1: No Delirium, 2: Non-hypoactive delirium, 3: Hypoactive delirium). ICU = intensive care unit, BCR = BUN/Creatinine ratio, APACHE = Acute Physiology and Chronic Health Evaluation, DMSS = Delirium Motor Subtype Scale.

**Table 2:** Logistic regression analyses.

	Univariate				Multivariate			
Variable	Non-Hypoactive Delirium		Hypoactive Delirium		Non-Hypoactive Delirium		Hypoactive Delirium	
	OR (95% CI)	*p*-Value	OR (95% CI)	*p*-Value	OR (95% CI)	*p*-Value	OR (95% CI)	*p*-Value
Age	1.03 (1.024–1.036)	<0.0001	1.032 (1.026–1.038)	<0.0001	1.025 (1.019–1.031)	<0.0001	1.024 (1.019–1.03)	<0.0001
Sex (female)	1.001 (0.848–1.18)	0.9941	1.148 (0.977–1.349)	0.0929	0.938 (0.79–1.112)	0.4599	1.04 (0.878–1.232)	0.6505
APACHE II	1.057 (1.048–1.067)	<0.0001	1.072 (1.062–1.081)	<0.0001	1.044 (1.034–1.055)	<0.0001	1.054 (1.044–1.064)	<0.0001
BCR	0.998 (0.993–1.004)	0.5224	1.011 (1.008–1.015)	<0.0001	0.995 (0.989–1.002)	0.1368	1.012 (1.008–1.016)	<0.0001
Kidney disease	1.738 (1.328–2.275)	<0.0001	1.984 (1.538–2.56)	<0.0001	1.114 (0.828–1.501)	0.4756	1.316 (0.987–1.754)	0.061
Diabetes mellitus	1.516 (1.172–1.961)	0.0015	1.555 (1.209–2.001)	0.0006	1.285 (0.98–1.687)	0.0701	1.227 (0.933–1.612)	0.1426
Heart disease	1.387 (1.048–1.836)	0.0223	1.344 (1.016–1.778)	0.0387	1.027 (0.761–1.385)	0.8621	0.978 (0.722–1.327)	0.8887
Stroke	1.934 (1.275–2.933)	0.0019	4.079 (2.961–5.619)	<0.0001	1.355 (0.875–2.098)	0.1735	2.63 (1.861–3.716)	<0.0001
Sepsis	2.258 (1.666–3.06)	<0.0001	3.162 (2.413–4.144)	<0.0001	1.674 (1.207–2.322)	0.002	2.016 (1.499–2.712)	<0.0001
Sedative use	1.322 (1.061–1.647)	0.013	1.174 (0.952–1.448)	0.1331	1.59 (1.236–2.047)	0.0003	1.464 (1.146–1.869)	0.0023
Surgical patients	0.75 (0.634–0.888)	0.0008	0.637 (0.541–0.751)	<0.0001	0.747 (0.608–0.917)	0.0054	0.765 (0.627–0.934)	0.0084
AUC (95% CI)					0.694		0.739	

APACHE = Acute Physiology and Chronic Health Evaluation, BCR = Blood urea nitrogen/creatinine ratio.

## Data Availability

The data in this study are available upon reasonable request from the corresponding author.
